# Dissecting Supplement and Nutrients Intake of Adults with and without COVID-19 History through the Lens of Health Belief Model

**DOI:** 10.3390/nu14214450

**Published:** 2022-10-22

**Authors:** Trias Mahmudiono, Cindra Tri Yuniar, Risti Kurnia Dewi, Qonita Rachmah, Dominikus Raditya Atmaka, Eurika Zebadia, Nur Sahila, Mutiara Arsya Vidianinggar Wijanarko, Chika Dewi Haliman, Shirley Gee Hoon Tang

**Affiliations:** 1 Department of Nutrition, Faculty of Public Health, Universitas Airlangga, Surabaya 60115, Indonesia; 2 Department of Pharmacology and Clinical Pharmacy, School of Pharmacy, Institut Teknologi Bandung, Bandung 40132, Indonesia; 3 Department of Nutrition, Faculty of Public Health, Andalas University, Padang 25175, Indonesia; 4 Department of Health Policy and Administration, Faculty of Public Health, Universitas Airlangga, Surabaya 60115, Indonesia; 5 Center for Toxicology and Health Risk Studies (CORE), Faculty of Health Sciences, Universiti Kebangsaan Malaysia, Jalan Raja Muda Abdul Aziz, Kuala Lumpur 50300, Malaysia

**Keywords:** health belief model, COVID-19, health well-being

## Abstract

Over the past two years, the world has faced the pandemic, COVID-19, and various changes. Several regulations and recommendations from the Ministry of Health of Indonesia have contributed to behavioral changes among Indonesian residents, especially in food consumption patterns. The change in food consumption patterns can be a positive change that formed due to the COVID-19 pandemic. This study aimed to examine whether the application of a Health Belief Model (HBM)-based nutrition education programme can be effectively used in changing the beliefs of adults with or without a COVID-19 history in supplement and nutrient intake. This study was a cross-sectional study involving 140 adults. This study placed 70 adults with/without a COVID-19 history into the intervention group. The intervention group participated in a nutrition education programme. The respondents were asked to fill out the questionnaire. The data were analyzed by independent and paired *t*-tests and Chi-square test. The result of this study showed no association between perceived susceptibility, severity, benefit, barrier, and self-efficacy, of nutrient and supplement intake with the history of COVID-19 among the respondents. However, most of the respondents in this study were low in their scores of perceivedness. Thus, it is still important for the government to increase nutrient and supplement intake education, especially in young adults aged below 25 years old.

## 1. Introduction

For the past two years, the whole world has been facing a pandemic, COVID-19. Globally, as of March 2022, the number of total cases was 471 million [1]. Meanwhile, the total number of COVID-19 cases in Indonesia was 5.96 million cases as of 21 March 2022 [2]. However, the number of those who survived or were able to recover was 407 million globally and 5.58 million nationally, up to this day (March 2022) [1,2]. This pandemic caused several changes in Indonesia, especially after the release of regulations on the new-normal and several recommendations from the Ministry of Health to increase immunity [3,4].

Since the first COVID-19 case was found in Indonesia, the importance of proper nutritious food as a requirement for a healthy immune system was widely emphasized. One of the recommendations from the Ministry of Health of Indonesia was to eat nutritious food or a balanced diet [4]. The consumption of vegetables and fruits increased by more than 35% during the pandemic situation among Indonesians [5]. Interestingly, fast-food consumption decreased by more than 50% during the lockdown situation [5]. There was also a shift in expenditure on food ingredients among Indonesians, showing that during the pandemic of COVID-19, Indonesians were more likely to spend their money on food ingredients or fresh food than instant or processed food and tobacco [6]. A similar study also verified previous findings that fresh product purchases (e.g., fresh vegetables, fresh fruit, eggs) increased during the COVID-19 pandemic, but consumption of carbonated drinks, energy drinks, syrups, ready-to-eat meals, and canned food, decreased [7]. In addition to a balanced diet, the Indonesian Ministry of Health also recommends consuming supplements when needed [4]. The consumption of supplements among Indonesians increased by more than 47% during the lockdown. A study on the Indonesian elderly population showed that most elders agreed that supplements are needed to protect them during the new-normal era [8]. Thus, these previous studies could suggest a shift in Indonesian consumption of fresh vegetables, fruits, and supplements, during the COVID-19 pandemic.

The increased consumption of fruits, vegetables, and supplements could become a positive behavior that formed during the pandemic. The consumption of fruits, vegetables, and supplements, not only served to prevent COVID-19 but also other non-communicable diseases related to nutrition that emerged in Indonesia, such as diabetes and hypertension [9]. This positive behavior could result in positive health outcomes if carried out continuously.

The health belief model (HBM) was a model that focused on efforts to improve public health by understanding why people failed to adopt preventive health measures [10]. This model has been successfully replicated countless times showing its ability to explain and predict various behaviors associated with positive health outcomes [11]. There are four individual perceptions that could predict their behavior [10]. The first one is perceived susceptibility. This perception argues that people will be more motivated to act on a healthy behavior if they believe they are susceptible to a negative health outcome. The second is perceived severity. This argues that the stronger people’s perception of the severity of negative health outcomes, the more they will be motivated to act to avoid that outcome. Third, perceived barriers and benefits that argue when strong barriers were found in adopting the preventive behavior, they will be unlikely to do so. Lastly, self-efficacy proposed that overall motivation to pursue health could affect their decision to present positive behavior.

The COVID-19 pandemic caused several changes in the quality of life (QoL) and the health-related quality of life (HR-QoL) of survivors [12,13]. Moreover, those who survived COVID-19 were experiencing different pressure and problems compared to those without a COVID-19 history. This could suggest different values, views, and especially perceptions, between those with and without a COVID-19 history towards preventive behavior. Furthermore, the HBM model constructed by perceived susceptibility, perceived severity, perceived barriers, and benefits, was found to be effective in increasing the perceived susceptibility and severity in patients or survivors to exhibit positive behavior [14]. Therefore, this study aimed to assess the influence of the HBM in nutrition education intervention to improve supplement and nutrient intake of adults with and without a COVID-19 history.

## 2. Materials and Methods

This intervention study uses a quasi-experimental design located in Surabaya, Padang, and Bandung. The sample size of this study was calculated using simple random sampling formula. According to that formula, the sample size of this study was 140 people. Thus, the number of those in control with/without a COVID-19 history and intervention with/without a COVID-19 history group was 35 per group. The inclusion criteria for this study were an adult (18–59 years old), with or without a history of COVID-19, who agreed to join the study by signing the informed consent.

The instrument used in collecting baseline data was a questionnaire. As for the implementation of education, three modules were developed as educational media. The three modules were entitled “Balanced Nutrition and Health Belief Model of the COVID-19 Pandemic”, “Nutrition and Immunity”, and “The Role of Supplements in Increasing Body Endurance”.

The intervention group received educational material once every two weeks for three months resulting in six meetings. The control group received education about “Clean and Healthy Living Behavior during the COVID-19 Pandemic”, a module by the Ministry of Education and Culture, Indonesia.

The dependent variable in this study was the education materials that were provided to the respondents. The independent variable in this study was the respondents’ perceived susceptibility and perceived severity.

Descriptive and inferential statistical analysis were conducted. The descriptive analysis determined the respondents’ characteristics. The inferential analysis was performed to determine the difference between each group’s perceived susceptibility and perceived severity. The paired sample *t*-test was applied to assess the difference between pre and post-test in each group. Meanwhile, the independent samples *t*-test was employed to assess the differences between each group.

## 3. Results

This study was conducted on 140 respondents with and without a COVID-19 history. The majority of the respondents were aged 18–25 years old (86%), with an age distribution of 18–60 years old. Their educational background was very diverse with the mean graduating from high school/equivalent, with 50% having a history of COVID-19, and 50% with-out a history of COVID-19. Most of the respondents with a history of COVID-19 1× were 41%. The anthropometric examination of respondents was very diverse, with a distribution of 145–190 cm in height and 30–120 kg in weight (Table 1).

### 3.1. Characteristics of the Respondents

#### 3.1.1. Perceived Susceptibility

Perceived susceptibility is an individual’s belief about susceptibility to the risk of disease in encouraging people to adopt healthier behaviors. The questions asked were about the relationship between eating a balanced nutritional diet and a person’s immune system, and immunity in the face of COVID-19. The following are the results of the distribution of responses from 140 respondents (Table 2).

From the table above, respondents who experienced COVID-19 (70%) agreed that if they do not eat balanced diets are at risk of being infected by COVID-19 and are more likely to get sick easily (85.7%). The respondents also agreed that everyone who does not consume staple food (rice, potatoes, corn) (48.5%), animal protein (62.8%), vegetable protein (60.0%), vegetables (72.8%), or fruit (72.8%) in sufficient quantities and drink eight glasses of water per day (60.0%) are at risk of being infected with COVID-19. Both respondents who have a COVID-19 history (94.2%), and who do not (90.0%), agreed that the risk of getting infected is increased if family members are affected by COVID-19. The respondents with a COVID-19 history (70.0%) agreed that if their family gets COVID-19 and they eat a balanced diet, they will not get infected easily. In addition, the respondents without a COVID-19 history agreed that they are at a very easy risk of being infected withCOVID-19. Both respondents who have a COVID-19 history (78.5%), and who do not (71.4%), agreed to seek treatment to reduce their exposure to COVID-19.

#### 3.1.2. Perceived Severity

Perceived severity or seriousness are the feelings about the seriousness of COVID-19, including evaluating clinical and medical consequences (for example, death, disability, and illness) and possible social consequences (such as effects on work, family life, and social relationships). Many experts combine the two components above as a perceived threat.

Based on Table 3 above, it was noticed that there was a difference in the percentage between respondents with a COVID-19 history (52.8%) and those without a COVID-19 history (65.7%) that agreed that if I get COVID-19 it can cause death. As for this study, respondents with (72.8%) and without (82.8%) COVID-19 agreed that they are worried about getting infected by COVID-19. This result was also the same as the next statement, where most respondents agreed that the COVID-19 pandemic made me overthink things like “Is there going to be a virus in this thing?”. Furthermore, respondents who agreed that “if the pandemic will still have next wave” were from those who had no history (67.1%).

#### 3.1.3. Perceived Benefit

Perceived benefit is the acceptance of a person’s susceptibility to a condition believed to cause seriousness (perceived threat) to encourage it to produce a force that supports changes in nutritional behavior. This depends on a person’s belief in the effectiveness of the various available efforts in reducing the threat of COVID-19, or the perceived benefits of taking these health efforts. When a person believes in susceptibility and seriousness, he/she is often not expected to accept any recommended health measures unless they are deemed appropriate.

The following are the results of the distribution of answers from 140 respondents. These questions are related to perceived benefit and are broken down into 10 questions about various spectrums related to balanced nutrition consumption (Table 4).

Based on Table 4, we found that there was a difference in the percentage between respondents with a COVID-19 history (94.3%) and those without a COVID-19 history (95.7%) that agreed if they eat food according to balanced nutrition guidelines, they will be healthier. The results showed that there was a higher percentage of respondents without a COVID-19 history (92.8%) who agreed that if they take supplements their immune system would be stronger, as compared to those who had a COVID-19 history (88.6%). However, this result was different from the next perceived benefit question, where most respondents with a COVID-19 history (91.4%) agreed that if they take supplements they will be healthier and fitter during the pandemic, compared to respondents who did not have a COVID-19 history (87.1%). Furthermore, respondents who agreed that if they took supplements they were more comfortable traveling were mostly respondents with a COVID-19 history, compared to those with no history. This result is also the same as the next statement where most respondents who agreed that taking supplements will be more confident and are mostly from the group of respondents with a COVID-19 history (75.7%), compared to those who do not have a history (61.4%). However, there is no difference in the percentage of respondents who agreed that eating foods according to balanced nutrition guidelines will have a more positive self-image between respondents with a COVID-19 history (84.3%) and those without a COVID-19 history (84.3%). The largest percentage of respondents who agreed that eating food according to balanced nutrition guidelines would increase their productivity came from respondents with a COVID-19 history (88.6%), compared to those without a COVID-19 history (84.3%). In addition, there was a difference between respondents with a COVID-19 history (74.3%) and without a COVID-19 history (72.3%) who stated that eating food according to balanced nutrition guidelines will increase their confidence. There was no difference observed in the percentage between respondents with a COVID-19 history (74.3%) and those without a COVID-19 history (74.3%) who agreed that eating a balanced nutritious diet will reduce their stress. However, it was found that most of the respondents without COVID-19 (88.6%) agreed that eating a balanced diet would prevent various non-communicable diseases when compared to those who had a COVID-19 history (85.7%).

#### 3.1.4. Perceived Barrier

Perceived barriers are potential negative aspects of a prevention and treatment effort in dealing with COVID-19 (such as uncertainty, and side effects), or perceived barriers (such as worrying about being unsuitable, unhappy, or nervous), which may serve as barriers to recommending a behavior. The following are the results of the distribution of answers from 140 respondents. These questions are related to perceived barriers and are broken down into 10 questions about various spectrums related to balanced nutrition consumption. The following elaboration described the related details of each question.

Based on the results in Table 5, we observed that most of the respondents who stated that they agreed that at the end of the month they could not eat a balanced nutritional diet because it was expensive, mostly came from the group of respondents who had a COVID-19 history (41.4%) compared to those without a COVID-19 history (34.3%). This result was the same as the next perceived barrier question where the majority of respondents with a COVID-19 history (48.6%) agreed that because they were busy working, they could not regulate their meal portions according to balanced nutrition guidelines compared to those without a COVID-19 history (44.3%). Additionally, there was a difference in the percentage between respondents with a COVID-19 history (27.1%) and without a COVID-19 history (17.1%) who agreed that they feel bad when they have to eat salad alone when friends eat fast food. The results of this study also found that there was a higher percentage of respondents with a COVID-19 history (8.0%) who agreed that they were opposed by their parents when they wanted to take supplements to boost their immunity, compared to those without a COVID-19 history (1.4%). Furthermore, most respondents who agreed that when they were on vacation they found it difficult to eat a balanced diet mostly came from respondents with a COVID-19 history (44.3%), compared to those without a COVID-19 history (24.3%). The present study also observed that the highest percentage who agreed that they could not control the portion of their meal when eating with their family came from the group of respondents with a COVID-19 history (52.3%), compared to those without a COVID-19 history (42.8%). It was also shown that respondents who agreed that they could not take supplementation due to certain diseases are mostly respondents without a COVID-19 history (5.7%), compared to those with a COVID-19 history (4%). Furthermore, respondents who stated that they did not take supplements because they did not know the type and dose to take were mostly from the group of respondents without a COVID-19 history (27.1%), compared to those with a COVID-19 history (20%). The group of respondents who stated that they were not familiar with the concept of balanced nutrition mostly came from the group of respondents without a COVID-19 history (20%), compared to those with COVID-19 history (10%). Likewise, respondents who agreed that balanced nutritional food could not be found in food delivery apps mostly came from respondents without a COVID-19 history (21.4%), compared to those with a COVID-19 history (20%).

#### 3.1.5. Self-Efficacy

Self-efficacy is a belief in a person’s ability to take an action related to balanced nutrition consumption and supplementation consumption to achieve a goal in preventing and dealing with the COVID-19 pandemic. The following are the results of the distribution of answers from 140 respondents. Question G is related to self-efficacy and it is broken down into 10 questions asking about various spectrums related to balanced nutrition consumption. The following elaboration described the related details of each question.

Based on Table 6, we discovered that 32.9% of the respondents who have experienced COVID-19 agreed with the statement that they can consume well-balanced nutritious food every day. On the other hand, 48.6% of respondents who have not experienced COVID-19 agreed that they have self-efficacy to consume well-balanced nutritious food daily. From question 1, we discovered that the percentage of respondents who agreed that they are able to consume well-balanced nutritious food every day was higher in those who have not experienced COVID-19 compared to those who have. From question 2, we discovered that 64.3% of the respondents who have experienced COVID-19 agreed with the statement that they can consume well-balanced nutritious food at least two times a day. On the other hand, 62.9% of respondents who have not experienced COVID-19 have the self-efficacy to consume well-balanced nutritious food two times a day. From question 2, we discovered that the percentage of respondents who agreed that they can consume well-balanced nutritious food two times a day was higher in those who have experienced COVID-19, compared to those who have not. From question 3, we observed that 58.6% of the respondents who have experienced COVID-19 agreed with the statement that they are able to consume well-balanced nutritious food on the weekend. On the other hand, 55.7% of respondents who have not experienced COVID-19 agreed that they have the self-efficacy to consume well-balanced nutritious food at the weekend. From question 3, we discovered that the percentage of respondents who agreed that they are able to consume well-balanced nutritious food at the weekend is higher in those who have experienced COVID-19, compared to those who have not. From question 4, it was found that 61.4% of the respondents who have experienced COVID-19 agreed with the statement that they are able to consume well-balanced nutritious food at least once a week. However, 55.7% of respondents who have not experienced COVID-19 agreed that they have the self-efficacy to consume well-balanced nutritious food once a week. From question 4, we revealed that the percentage of respondents who agreed that they are able to consume well-balanced nutritious food once a week was higher in those who have ever experienced COVID-19, compared to those who have not. From question 5, we demonstrated that 55.7% of the respondents who have experienced COVID-19 agreed with the statement that they are able to consume well-balanced nutritious food once in two weeks. On the other hand, 51.4% of respondents who have not experienced COVID-19 agreed that they have the self-efficacy to consume well-balanced nutritious food once in two weeks. From question 5, we showed that the percentage of respondents who agreed that they are able to consume well-balanced nutritious food once in two weeks was higher in those who have never experienced COVID-19, compared to those who have not. From question 6, it was noticed that 54.2% of the respondents who have experienced COVID-19 agreed with the statement that they are able to consume well-balanced nutritious food even if it is expensive. However, 48.6% of respondents in this study who have not experienced COVID-19 agreed that they have the self-efficacy to consume well-balanced nutritious food even if it is expensive. From question 6, we observed the percentage of respondents who agreed that they are able to consume well-balanced nutritious food even if it is expensive was higher in those who have experienced COVID-19, compared to those who have not. From question 7, we examined that 41.4% of the respondents who have experienced COVID-19 agreed with the statement that they are able to consume well-balanced nutritious food while travelling. On the other hand, 48.6% of respondents who have not experienced COVID-19 have the self-efficacy to consume well-balanced nutritious food while travelling. From question 7, we concluded that the percentage of respondents who agreed that they are able to consume a well-balanced nutritious diet while travelling was higher in the respondents who have not experienced COVID-19, compared to those who have. From question 8, we found that 51.4% of respondents who have ever experienced COVID-19 agreed that they have the self-efficacy to consume a well-balanced nutritious diet still while being away from home. On the other hand, 55.7% of respondents who have not experienced COVID-19 agree that they will still consume a well-balanced diet even when they are away from home. From question 8, we revealed that the percentage of agreement with the statement of self-efficacy related to the resilience of well-balanced nutritious food consumption away from home was higher in those respondents who have not experienced COVID-19, compared to those who have. From question 9, we demonstrated that there were 35.7% of respondents who have experienced COVID-19 agreed that they have self-efficacy to still consume well-balanced nutritious food during hustle. However, 32.8% of respondents who have not experienced COVID-19 agreed that they will still consume well-balanced nutritious food during hustle. From question 9, we conclude that the percentage of agreement with the statement of self-efficacy related to the resilience of well-balanced nutritious consumption during hustle is higher in those respondents who have experienced COVID-19, compared to those who have not. From question 10, we examined that 38.6% of respondents who have experienced COVID-19 agreed that they have self-efficacy to still consume a well-balanced nutritious food even if it is far from their board. On the other hand, 30% of respondents who have not experienced COVID-19 agreed that they will still consume a well-balanced nutritious diet even if it is away from their board. From question 10, we concluded that the per-centage of agreement with the statement of self-efficacy related to the resilience of well-balanced nutritious food consumption even if it is less accessible was higher in those who have experienced COVID-19, compared to those who have not.

#### 3.1.6. Kessler Psychological Distress Scale (K10)

The Kessler Psychological Distress Scale is a 10-item questionnaire intended to produce a global measure of distress based on questions about anxiety and depressive symptoms that a person has experienced in the past four weeks, in the face of the COVID-19 pandemic. The following are the results of the distribution of answers from 140 respondents.

Based on Table 7, we want to explain questions 1 to 10 related to psychological distress results among the respondents. From question 1, we examined that 30% of the respondents who have experienced COVID-19 agreed with the statement that they ever feel exhausted without clear reason. On the other hand, 25.7% of respondents who have not experienced COVID-19 agreed that they ever feel exhausted without clear reason. From question 1, we found that the percentage of respondents who agreed that they feel exhausted without a clear reason was higher in those who have ever experienced COVID-19. From question 2, we showed that 15.7% of the respondents who have experienced COVID-19 agreed that they feel a lot of stress. However, 12.8% of respondents who have not experienced COVID-19 agreed that they feel a lot of stress. From question 2, we found that the percentage of respondents who agreed that they feel a lot of stress was higher in those who have ever experienced COVID-19, compared to those who have not. From question 3, we revealed that 20% of the respondents who have experienced COVID-19 agreed with the statement that they feel stress until they cannot relax. On the other hand, 14.3% of the respondents who have not experienced COVID-19 agreed that they feel stressed until they cannot relax. From question 3, it was observed that the percentage of respondents who agreed that they feel stress until they cannot relax was higher in those who have experienced COVID-19, compared to those who have not. From question 4, we demonstrated that 11.4% of the respondents who have experienced COVID-19 agreed with the statement that they feel hopeless, whereas 18.6% of the respondents who have not experienced COVID-19 agreed that they feel hopeless. From question 4, we showed that the percentage of respondents who agreed that they feel hopeless was higher in those who have not experienced COVID-19, compared to those who have. From question 5, it was noticed that 21.4% of the respondents who have experienced COVID-19 agreed that they ever feel nervous or anxious. A total of 25.7% of the respondents who have not experienced COVID-19 agreed that they feel nervous or anxious. From question 5, we found that the percentage of respondents who agreed that they feel nervous or anxious was higher in those who have not experienced COVID-19, compared to those who have. From question 6, we observed that 14.3% of the respondents who have experienced COVID-19 agreed that they feel anxious until they cannot sit quietly. However, only 10% of the respondents who have not experienced COVID-19 agreed that they feel anxious until they cannot sit quietly. From question 6, researchers concluded that the percentage of respondents who agreed that they feel anxious until they cannot sit quietly was higher in those who have experienced COVID-19, compared to those who have not. From question 7, we examined that 17.1% of the respondents who have experienced COVID-19 agreed that they feel depressed. On the other hand, 21.4% of the respondents who have not experienced COVID-19 agreed that they feel depressed. From question 7, we showed that the percentage of respondents who agreed that they feel depressed was higher among those who have not experienced COVID-19, compared to those who have. From question 8, we revealed that 37.1% of the respondents who have experienced COVID-19 agreed that they need effort before doing something. However, 31.4% of the respondents who have not experienced COVID-19 agreed that they need effort before doing something. From question 8, we examined that the percentage of respondents who agreed they need effort before doing something was higher in those who have experienced COVID-19 than those who have not. From question 9, it was found that 18.6% of the respondents who have experienced COVID-19 agreed that they feel sad until nothing can cheer them up. On the other hand, 21.4% of the respondents who have not experienced COVID-19 agreed that they feel sad until nothing can cheer them up. From question 9, we concluded that the percentage of respondents who agreed that they feel sad until nothing can cheer them up was higher in those who have not experienced COVID-19, compared to those who have. From question 10, we examined that 12.8% of the respondents who have experienced COVID-19 agree that they feel worthless. On the other hand, 14.3% of the respondents who have not experienced COVID-19 agreed that they feel worthless. From question 10, we showed that the percentage of respondents who agreed that they feel worthless was higher among those who have not experienced COVID-19, compared to those who have.

#### 3.1.7. Emotional Eating Questionnaire

The Emotional Eating Scale (EES) consists of four dimensions, namely: Anger (anger), Anxiety (anxiety), Depression (depression), and Somatic (somatic). This measuring tool consists of 10 items that can describe the four dimensions. A total of two items describes the Anger dimension (anger), four items describe the Anxiety dimension (anxiety), two items describe the Depression dimension (depression), and two other items describe the Somatic dimension (somatic). The following are the results of the distribution of answers from 140 respondents (Table 8).

From the table above, we know that in question 1 the majority answered disagree to change their mood due to weight movement (61.4%), but on the other hand, 38.5% of the participants felt weight distortion of their body affected their mood production. The questions about craving food might be felt by 55% of total respondents who answered some-times and often eat certain foods. The phenomenon of sugary food declined by 65% of participants who did not agree with the statement that it is difficult to stop consuming sweet foods, such as chocolate. Question number 4 was answered by 67% of participants who did not have any problem controlling the number of certain foods they consume. Despite that, 32.1% had some problems getting on their daily diet. Question number 5 presented any kind of food which they consume when they are stressed, angry, or bored. A total of 50% answered that it is a necessary thing to eat some food when they do not feel well. Question number 6 proves that 50.7% of respondents that eat more favorites became troubled controlling them. Question number 7 shows that 56.4% of participants did not feel guilty if they eat foods that they are forbidden to eat, such as sweet foods or snacks. Less control of the food when they feel tired after work is felt by 49.28% of total respondents. Question number 9 talks about people who feel like giving up on food control, and eating without control, which was answered by 25.7% of the participants, and 74.28% still feel secure to maintain what they eat.

#### 3.1.8. Association

Most of the respondents in our study scored low in perceived susceptibility, severity, benefit, barrier, and self-efficacy. There was no association between perceived susceptibility, severity, benefit, barrier, and self-efficacy, with the history of COVID-19.

Table 9 shows that there is no significant association between low perceived susceptibility and COVID-19 infection. The insignificant association is shown by the *p* value > 0.05. The insignificance association also happens to the other independent variables, such as average perceived susceptibility, low perceived severity, average perceived severity, low perceived benefit, average perceived benefit, low perceived benefit, low perceived barrier, average perceived barrier, low self-efficacy, and average self-efficacy with the incidence of COVID-19 infection. However, the independent variable of low perceived benefit shows that those with low perceived benefit are 1926 times more likely to experience COVID-19. Another variable, which is average perceived benefit, shows that those with average perceived benefit are 1008 more likely to experience COVID-19. Low-perceived barrier respondents are also 1222 times more likely to be infected by COVID-19. The last independent variable, the average perceived barrier, shows that those respondents with an average perceived barrier are 1545 times more likely to be infected by COVID-19.

## 4. Discussion

Perceived susceptibility is an individual’s belief about his susceptibility to the risk of disease in encouraging people to adopt healthier behaviors [15]. The questions asked were about the relationship between eating a balanced nutritional diet and a person’s immune system, and immunity in the face of COVID-19. In this study, most respondents had low and average scores of perceived susceptibilities. This could be due to poor knowledge [16].

Perceived severity or seriousness that is felt is the feelings about the seriousness of COVID-19, include evaluating clinical and medical consequences (for example, death, disability, and illness), and possible social consequences (such as effects on work, family life, and social relationships) [17]. Many experts combine the two components above as a perceived threat. In this study, most respondents had a low score of perceived severity.

Perceived benefits is the acceptance of a person’s susceptibility to a condition that is believed to cause seriousness (perceived threat), to encourage it to produce a force that supports changes in nutritional behavior [18]. This depends on one’s belief in the effectiveness of the various available efforts in reducing the threat of COVID-19, or the perceived benefits of taking these health efforts. When a person believes in susceptibility and seriousness, he/she is often not expected to accept any recommended health measures unless they are deemed appropriate.

Perceived barriers or perceived barriers to change [15], the potential negative aspects of a prevention and treatment effort in dealing with COVID-19 (such as uncertainty, and side effects), or perceived barriers (such as worrying about being unsuitable, unhappy, or nervous), may serve as barriers to recommending a behavior. One of the factors related to perceived barrier is the lack of knowledge among the respondents. A recent study conducted by Birihane et al. [19] showed that there was a relationship between the knowledge of the health care providers and their actions to prevent COVID-19. The study found that the respondents who have perceived barriers were more likely to not execute procedures related to COVID-19 prevention.

Self-efficacy is a belief in a person’s ability to take an action to achieve a goal in preventing and dealing with the COVID-19 pandemic [15]. In this study, most of the respondents had low self-efficacy scores. This showed that there was a lack of belief among the respondents to take an action, especially in providing good nutrients and supplements to prevent and deal with COVID-19. Self-efficacy is one of the factors associated with the ability of individuals to control their emotions, as well as being implicated with a lower risk of anxiety. Recently, Delshad et al. [20] reported that the enhancement of self-efficacy level might reduce anxiety. This is because people who have high self-efficacy levels can employ more different ways to achieve their goals, hence, positively increase a person’s ability to cope with stress and reduce anxiety in dealing with the COVID-19 pandemic. Additionally, Meyer et al. [21] also revealed that enhancing self-efficacy was determined to be the most important element in coping with high levels of perceived COVID-19 related stress.

According to our knowledge, this is the first study assessing adults’ perceived nutrient and supplement intake among adults in Surabaya, Indonesia. There were several limitations in this study including potential selection bias, self-reported data, and recall bias. Selection bias might arise from the recruitment of respondents by the enumerator, which seems to have a higher response rate among young adult population. Even though, currently, Indonesia is undergoing a demographic dividend, the generalization of our study result to the whole population of Surabaya, Indonesia, should be made with caution. In order to minimize recall bias and measurement bias, we employed trained enumerators to guide respondents to go through the questionnaire, using probing question if necessary.

## 5. Conclusions

According to the construct of the Health Belief Model, the overall score of perceived susceptibility, severity, benefit, barrier, and self-efficacy, showed no significant association with the history of COVID-19 among young adults. However, association between question items of all variables in the construct of the Health Belief Model showed significant results. Thus, it is still important for the government to increase socialization and education on the importance of nutrients and supplement intake to protect themselves, and to prevent them from being infected with COVID-19.

## Figures and Tables

**Table 1:** Characteristics of the respondents.

Characteristics	Category	(*n*)	%
Age	18–25 years old	120	85.71
	26–30 years old	7	5.00
	31–25 years old	3	2.14
	36–40 years old	3	2.14
	41–45 years old	2	1.42
	46–50 years old	1	0.71
	51–55 years old	1	0.71
	56–60 years old	3	2.14
Education Level	Uneducated	3	2.14
	Primary school graduated	1	0.71
	Secondary school graduated	1	0.71
	High school graduate	90	64.28
	Diploma	18	12.85
	University graduated	27	19.28
COVID-19 History	Yes	70	50.00
	No	70	50.00
Frequency Being Infected by COVID	Never	70	50.00
	Once	57	40.71
	Twice	11	7.85
	Three times	2	1.42
Treatment during COVID-19	Isolation at home	78	55.71
	Isolation at hotel	25	17.85
	Isolation in healthcare	12	8.57
	Isolation at hospital	26	18.57
Weight	30–40 kg	7	5.00
	41–50 kg	43	30.71
	51–60 kg	47	33.57
	61–70 kg	28	20.00
	71–80 kg	8	5.71
	81–90 kg	4	2.85
	91–100 kg	1	0.71
	101–110 kg	1	0.71
	111–120 kg	1	0.71
Height	145–150 cm	20	14.28
	151–160 cm	80	57.14
	161–170 cm	28	20.00
	171–180 cm	11	7.85
	181–190 cm	1	0.71

**Table 2:** Perceived susceptibility results.

Perceived Susceptibility Questions	Percentage of Respondents with Agreement		*p* Value
	With COVID-19 History *n* (%)	Without COVID-19 History *n* (%)	
Everyone who does not eat a balanced diet is at risk of infected COVID-19.	44 (62.8)	49 (70)	0.037
Everyone who does not eat a balanced diet gets sick easily.	61 (87.1)	60 (85.7)	0.01
Everyone who does not eat staple food (rice, potatoes, corn) in sufficient quantities is at risk of infected COVID-19.	34 (48.5)	32 (45.7)	0.049
Everyone who does not consume adequate amounts of animal protein is at risk of infected COVID-19.	44 (62.8)	41 (58.5)	0.05
Everyone who does not consume adequate amounts of vegetable protein is at risk of infected COVID-19.	38 (54.2)	42 (60)	0.021
Everyone who does not eat a sufficient amount of vegetables is at risk of infected COVID-19.	47 (67.1)	51 (72.8)	0.039
Everyone who does not eat the fruit in sufficient quantities is at risk of infected COVID-19.	51 (72.8)	49 (70)	0.04
Everyone who does not consume 8 glasses of water per day is at risk of infected COVID-19.	38 (54.2)	42 (60)	0.028
Because my family members are affected by COVID-19, my risk of getting infected are increased.	66 (94.2)	63 (90)	0.00
If my family gets COVID-19 and I eat a balanced diet then I won’t get infected easily.	45 (64.2)	49 (70)	0.001
I am a very easy risk of infected COVID-19.	27 (38.5)	18 (25.7)	0.005
I will seek treatment to reduce my exposure to the COVID-19.	55 (78.5)	50 (71.4)	0.002
I am at risk of having difficulty recovering from COVID-19.	5 (7.1)	7 (10)	0.03
It is possible that I will be susceptible to COVID-19 if my nutritional needs are not met.	53 (75.7)	56 (80)	0.036
It is likely that I will be malnourished if I have COVID-19 for a long time.	41 (58.5)	48 (68.5)	0.001
I will stay healthy even if my nutritional needs are not met	14 (20)	7 (10)	0.012
I will not be infected with COVID-10 even if my nutritional needs are not met.	7 (10)	13 (18.5)	0.05
I don’t care about the presence of COVID-19 and I continue to do my daily activities.	14 (20)	8 (11.4)	0.018

**Table 3:** Perceived severity results.

Perceived Severity Questions	Percentage of Respondents with Agreement		*p* Value
	With COVID-19 History *n* (%)	Without COVID-19 History *n* (%)	
If I get COVID-19 it can cause death.	37 (52.8)	46 (65.7)	0.051
I am worried that my family and I will be infected by COVID-19.	51 (72.8)	58 (82.8)	0.001
The COVID-19 pandemic makes me overthink things like “Is there going to be a virus in this thing?”.	43 (61.4)	42 (60)	0.034
If I get COVID-19, then I can’t do the things I like.	42 (60)	44 (62.8)	0.011
I believe the COVID-19 pandemic will still have the next wave.	41 (58.5)	47 (67.1)	0.000
If I get COVID-19, my family will not take care of me.	20 (28.5)	15 (21.4)	0.020
If I get COVID-19, then I will get a negative stigma from my neighbors and the environment where I live.	32 (45.7)	27 (38.5)	0.033
If I get COVID-19, then I lose my job.	12 (17.1)	12 (17.1)	0.001
If I get COVID-19, it can cause my income decrease.	30 (42.8)	37 (52.8)	0.032
If I get COVID-19, it can stress me out because I am too afraid to think about it.	32 (45.7)	41 (58.5)	0.048

**Table 4:** Perceived benefit results.

Perceived Benefits Questions	Percentage of Respondents with Agreement		*p* Value
	With COVID-19 History *n* (%)	Without COVID-19 History *n* (%)	
If I eat food according to balanced nutrition guidelines, I will be healthier.	66 (94.3)	67 (95.7)	0.011
If I take supplements, I feel my immune system is stronger.	62 (88.6)	65 (92.8)	0.002
If I take supplements, I feel healthier and fitter during the pandemic.	64 (91.4)	61 (87.1)	0.003
If I take supplements, I feel more comfortable to traveling.	55 (78.6)	46 (65.7)	0.092
If I take supplements, I feel more confident.	53 (75.7)	43 (61.4)	0.016
If I eat food according to balanced nutrition guidelines, my self-image changes in a more positive direction.	59 (84.3)	59 (84.3)	0.031
If I eat food according to balanced nutrition guidelines, I feel my productivity will increases.	62 (88.6)	59 (84.3)	0.004
If I eat a balanced nutritious diet, my self-confidence increases.	52 (74.3)	51 (72.3)	0.003
If I eat a balanced nutritious diet, I feel less stressed.	52 (74.3)	52 (74.3)	0.002
If I eat a balanced diet, I will be prevented from non-communicable diseases such as diabetes mellitus, hypertension, stroke, and others.	60 (85.7)	62 (88.6)	0.000

**Table 5:** Perceived barrier results.

Perceived Barrier Questions	Percentage of Respondents with Agreement		*p* Value
	With COVID-19 History *n* (%)	Without COVID-19 History *n* (%)	
If today is the end of the month, I can’t eat balanced nutritious food because it’s expensive.	29 (41.4)	24 (34.3)	0.044
I’m busy working so I can’t adjust the portion of food according to the recommended balanced nutrition.	34 (48.6)	31 (44.3)	0.051
I feel bad if I have to eat salad alone when my friends eat fast food.	19 (27.1)	12 (17.1)	0.05
I was opposed by my parents when I wanted to take supplements to boost my immunity.	6 (8)	1 (1.4)	0.022
I have a hard time eating a balanced nutritious diet when I’m on vacation.	31 (44.3)	24 (34.3)	0.029
I feel bad for refusing food so I can’t adjust the portion sizes according to balanced nutrition guidelines when I’m eating with my family.	37 (52.3)	30 (42.8)	0.000
I can’t take supplements because I have a certain disease.	3 (4)	4 (5.7)	0.001
I don’t know the type and amount of supplements I should take.	14 (20)	19 (27.1)	0.021
I am not familiar with the concept of balanced nutrition.	10 (10)	14 (20)	0.039
Nutritious balanced meals can’t be found in food delivery apps.	14 (20)	15 (21.4)	0.041

**Table 6:** Self-efficacy results.

Self Efficacy Questions	Percentage of Respondents with Agreement		*p* Value
	With COVID-19 History *n* (%)	Without COVID-19 History *n* (%)	
I am able to consume well balanced nutritious food every day.	23 (32.9)	34 (48.6)	0.003
I am able to consume well balanced nutritious food 2 times a day in minimum.	45 (64.3)	44 (62.9)	0.011
I am able to consume well balanced nutritious food in the weekend.	41 (58.6)	39 (55.7)	0.023
I am able to consume well balanced nutritious food at least once in a week.	43 (61.4)	39 (55.7)	0.034
I am able to consume well balanced nutritious food every 2 weeks.	39 (55.7)	36 (51.4)	0.05
I am able to consume well balanced nutritious food even though it is expensive.	38 (54.2)	34 (48.6)	0.001
I am able to consume well balanced nutritious food while traveling.	29 (41.4)	34 (48.6)	0.033
I am able to consume well balanced nutritious food even when I was not in my home.	36 (51.4)	39 (55.7)	0.048
I am able to consume well balanced nutritious food during hustle.	25 (35.7)	23 (32.8)	0.05
I am able to consume well balanced nutritious food even it is far from my board.	27 (38.6)	21 (30)	0.05

**Table 7:** Kessler psychological distress scale results.

Kessler Psychological Distress Scale Questions	Percentage of Respondents with Agreement		*p* Value
	With COVID-19 History *n* (%)	Without COVID-19 History *n* (%)	
Did you feel exhausted without clear reason?	21 (30)	18 (25.7)	0.002
Did you feel a lot of tension/stress?	11 (15.7)	9 (12.8)	0.01
Did you feel very stress thus you can not relax?	14 (20)	10 (14.3)	0.032
Did you feel hopeless?	8 (11.4)	13 (18.6)	0.038
Did you feel anxious and nervous?	15 (21.4)	18 (25.7)	0.001
Did you feel anxious until you can sit quietly?	10 (14.3)	7 (10)	0.002
Did you feel depressed?	12 (17.1)	15 (21.4)	0.031
Did you feel you need a lot of effort to do something?	26 (37.1)	22 (31.4)	0.005
Did you feel sad until nothing can cheer you up?	13 (18.6)	15 (21.4)	0.0092
Did you feel worthless?	9 (12.8)	10 (14.3)	0.052

**Table 8:** Emotional eating questionnaire results.

Questions	Disagree *n* (%)	Agree *n* (%)
Does your weight affect or changes your mood?	86 (61.4)	54 (38.5)
Do you have cravings for certain specific foods?	77 (55)	67 (47.1)
Is it difficult for you to stop consuming sweet foods, such as chocolate?	91 (65)	49 (35)
Do you have a problem controlling the number of certain foods you eat?	95 (67.8)	45 (32.1)
Is there anything to eat when you are stressed, angry, or bored?	70 (50)	70 (50)
Do you eat more of your favorite foods and have trouble controlling them?	69 (49.28)	71 (50.7)
Do you feel guilty if you eat foods that are forbidden to eat, such as sweet foods or snacks?	79 (56.4)	61 (43.5)
Do you feel less in control of the food you eat when you feel tired after work?	71 (50.7)	69 (49.28)
When you overeat, do you feel like giving up on food control and eating without control, especially foods that make you fat?	104 (74.28)	36 (25.7)
How often do you feel that food has control over you, rather than you controlling what you eat?	85 (60.7)	55 (39.28)

**Table 9:** Association between the variable results.

	*p* Value	OR	95% CI	
			Lower	Upper
Low Perceived Susceptibility	0.188	0.410	0.109	1.547
Average Perceived Susceptibility	0.551	0.717	0.241	2.137
Low Perceived Severity	0.121	0.411	0.134	1.265
Average Perceived Severity	0.257	0.554	0.199	1.539
Low Perceived Benefit	0.279	1.926	0.588	6.310
Average Perceived Benefit	0.988	1.008	0.375	2.705
Low Perceived Barrier	0.676	1.222	0.478	3.124
Average Perceived Barrier	0.344	1.545	0.628	3.801
Low Self Efficacy	0.507	0.692	0.234	2.050
Average Self Efficacy	0.515	0.727	0.278	1.900

## Data Availability

The datasets used and/or analyzed during the current study are available from the corresponding author upon reasonable request.
